# Neoaortic Regurgitation in Patients with Transposition Long Term After an Arterial Switch Operation and Its Relation to the Root Diameters and Surgical Technique Used

**DOI:** 10.1007/s00246-019-02217-w

**Published:** 2019-10-25

**Authors:** Krzysztof W. Michalak, Katarzyna Sobczak-Budlewska, Jacek J. Moll, Konrad Szymczyk, Jadwiga A. Moll, Monika Łubisz, Maciej Moll

**Affiliations:** 1 Department of Cardiology, Research Institute, Polish Mother’s Memorial Hospital, ul. Rzgowska 281/289, 93-338 Lodz, Poland; 2 Department of Cardiac Surgery, Polish Mother’s Memorial Hospital, Lodz, Poland; 3 Department of Diagnostic Imaging, Medical University of Lodz, Lodz, Poland

**Keywords:** Transposition of the great arteries, Arterial switch operation, Root dilatation, Neoaortic insufficiency

## Abstract

Neoaortic regurgitation and root dilatation are common findings in patients with transposition after an arterial switch operation. The aim of this study was to describe the relation between neoaortic regurgitation long term after an arterial switch procedure, aortic root diameters, and surgical technique used. We also assessed the agreement of the neoaortic regurgitation grade and root diameters in different imaging modalities. For this retrospective study, we qualified 56 consecutive patients who, according to our institutional protocol, had a routine postoperative evaluation of more than 16 years with multimodality imaging studies. Neoaortic regurgitation was assessed by both transthoracic echocardiography and magnetic resonance imaging, and the root diameters obtained by echocardiography and tomography were compared to the reference values and associated with the presence of neoaortic insufficiency. Neoaortic insufficiency was present in 75% of examined patients; the vast majority of them had trace or mild regurgitation, and its qualitative evaluation was significantly different between echocardiography and magnetic resonance imaging. In our study group, the neoaortic valve and aortic sinus were larger in relation to the normal values, and they were significantly correlated with the presence of neoaortic insufficiency, but not with the surgical technique used. Values obtained by echocardiography and tomography correlated well but were significantly different. Transthoracic echocardiography has a tendency to overestimate the severity of regurgitation compared to magnetic resonance imaging. Neoaortic valve and sinus dilatation are significantly correlated with valve insufficiency, but in most cases of root dilatation, the valve remains competent.

## Introduction

**Fig. 1:**
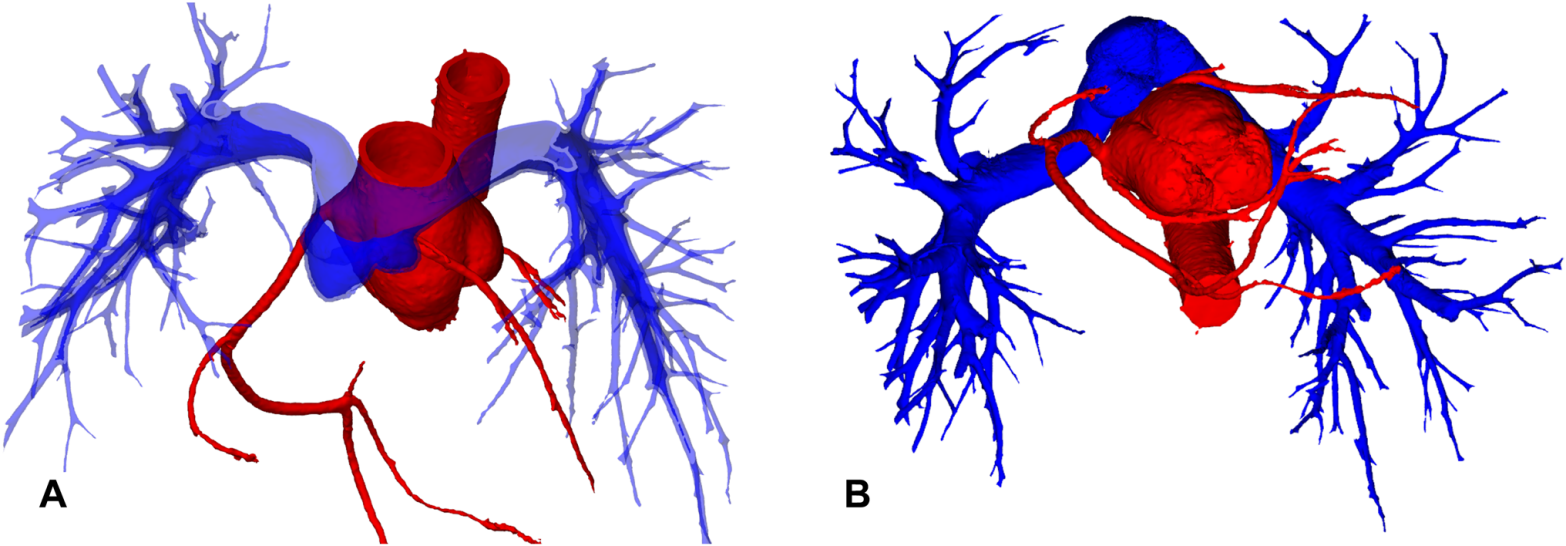
Volume-rendered 3D reconstruction of CT images. **a** Spatial relationship between the ascending aorta, main pulmonary artery, and pulmonary arteries. **b** Configuration of the aortic root and coronary arteries in relation to the main pulmonary artery

Neoaortic regurgitation (NeoAR) is a common sequela affecting patients with the transposition of the great arteries (TGA) after an arterial switch operation (ASO) [1]. In most cases, NeoAR usually does not constitute a significant clinical problem, but some patients still require reoperation because the insufficiency tends to progress [2, 3]. The reason for the common occurrence of neoaortic valve (NeoAoV) incompetence is multifactorial. First, it is a native pulmonary valve with less fibrous tissue supporting the valve annulus than the native aortic valve. There are also reported histological differences that make the pulmonary valve less resistant to the systemic circulation than the aortic valve [4]. The second major reason for the frequent occurrence of NeoAR seems to be dilatation and deformation of the neoaortic root. Initially, the native pulmonary valve is also wider than the aortic valve, especially in the presence of associated ventricular septal defect (VSDs) or the Taussig Bing anomaly. Additionally, implantation of the coronary arteries, connection to the ascending aorta, and bifurcation of the pulmonary arteries overriding the ascending aorta make the shape of the aortic root special and definitely different from the normal anatomy (Fig. 1a, b). The spatial configuration of the great vessels is important not only for root dilatation and valve regurgitation, but in some defined cases, it may also interfere with postoperative changes in the coronary artery anatomy, especially in cases of unfavorable features, such as proximal acute angulation and interarterial courses.

Because of the complex aortic root geometry, its detailed description is frequently difficult in echocardiography, especially in older patients. In our institution, as part of the routine follow-up protocol after an ASO for all patients who are older than 16 years, we perform standard transthoracic echocardiography (TTE), phase-contrast MRI, and coronary computed tomography (CT) angiography [5]. This multimodal approach is used for exact evaluation of the coronary pattern, arterial valve function, root diameters, and flow at different levels.

The aim of this study was to describe the relation between neoaortic regurgitation long term after an arterial switch procedure, aortic root diameters, and surgical technique used. We also assessed the agreement of the neoaortic regurgitation grade and root diameters in different imaging modalities.

## Patients and Methods

### Study Group

In our Institution between the years 1991 and 2018, 806 patients underwent an arterial switch operation. For this retrospective study, the initial cohort of 53 consecutive patients who had routine follow-up examinations late after an ASO by transthoracic echocardiography, MRI, and CT with a coronary artery evaluation and detailed measurements of the neoaortic root. Additionally, we included 3 patients who underwent all these studies earlier because of severe aortic regurgitation requiring precise assessment before surgical qualification. The study was approved by the institutional ethics board.

### Surgical Technique

The surgical technique was described previously [5, 6], here, we want to mention the important aspects for this study group. All switch operations were performed by one surgical team led by JJM. For all coronary transfers, we currently use the modified trap door technique, which was introduced in our institution in 1996; previously, the punch technique was used. The vast majority of our ASOs are performed with direct anastomosis of the major pulmonary artery. To facilitate that approach, the aorta is transected high above the valve and pulmonary trunk just above the commissures. This provides enough tissue to cover the holes in the neopulmonary sinuses created by harvesting the coronary artery, and the suture line is sufficiently long to prevent stenosis. It also shifts the aortic arch backwards, minimizing the risk of coronary artery compression by the major pulmonary artery or its branches.

### Data Analysis

All patients underwent a full TTE examination with an evaluation of NeoAR on a 5-grade scale (none, trace, mild, moderate, and severe) based on “eyeballing,” the diameter of the vena contracta, the ratio between the regurgitant jet and the left ventricular outflow tract (LVOT) diameter, reverse flow in the descending and abdominal aorta, the pressure half-time and the left ventricular diastolic diameter and volume. All studies yielded detailed measurements of the neoaortic root along the long parasternal axis (modified according to the anatomy of the ascending aorta) at the level of the hinge points of the valve cusps (neoaortic valve), aortic sinus, and sinotubular junction. All measurements were made according to the European Society of Cardiology (ESC) guidelines along the long parasternal axis, including measurements of the aortic annulus at peak systole and the aortic sinus and sinotubular junction at end-diastole [7]. The measurements were standardized to the patient height and weight using echocardiographic *z*-scores [8].

The CT examination of all patients provided a detailed description of the coronary anatomy with a major focus on the proximal parts, as well as a description of the aortic valve anatomy and aortic root measurements in two perpendicular planes at the level of the valve, aortic sinus, sinotubular junction, and mid-ascending aorta. All measurements were standardized to the patient height and width using the mean value of two perpendicular diameters and calculated *z*-scores for the tomographic data [9].

All MRI studies provided the diastolic volume of the left ventricle and aortic valve regurgitant flow calculated from the velocity-encoded phase-contrast sequence (electrocardiography gated, breath hold) with measurements performed in diastole at the level of the NeoAoV perpendicular to the LVOT. We evaluated the quantitative regurgitant fraction and the qualitative neoaortic insufficiency on a 5-grade scale: none, trace (regurgitant fraction: 0–8%), mild (8.1–20%), moderate (20.1–40%), and severe (> 40%) [10].

All results regarding NeoAR and aortic root diameters were correlated with potential risk factors, such as associated defects (VSDs, aortic arch anomalies, the Taussig Bing anomaly, coronary anomalies, bicuspid aortic valve, nonfacing commissures, and discrepancies between native aortic and pulmonary valves) or the surgical technique (trap door versus punch technique, direct pulmonary anastomosis versus patch reconstruction).

### Statistical Analysis

Statistical analyses were performed using Statistica 13 software (StatSoft, Inc., Tulsa, OK, USA). Quantitative data are presented as the mean with the 95% confidence interval (95% CI) and standard deviation (SD) or the median and interquartile range (IQR; 25th to 75th percentile) depending on the normality of the distribution. Qualitative data are presented as percentages. Associations between quantitative datasets were tested using Spearman’s or Pearson’s correlation test. Differences between the groups were assessed using the Mann–Whitney *U* test and ANOVA Kruskal–Wallis test or Student’s *t* test and ANOVA. The test selection was based on the normality of the data distribution. To evaluate the statistical relation between qualitative datasets, the *χ*^2^ test or Fisher exact test was used. The agreement between diameters obtained by two different methods was assessed by correlation tests and paired tests (*t* test or Wilcoxon test). A *p* value less than 0.05 was considered statistically significant.

## Results

### Neoaortic Regurgitation

**Fig. 2:**
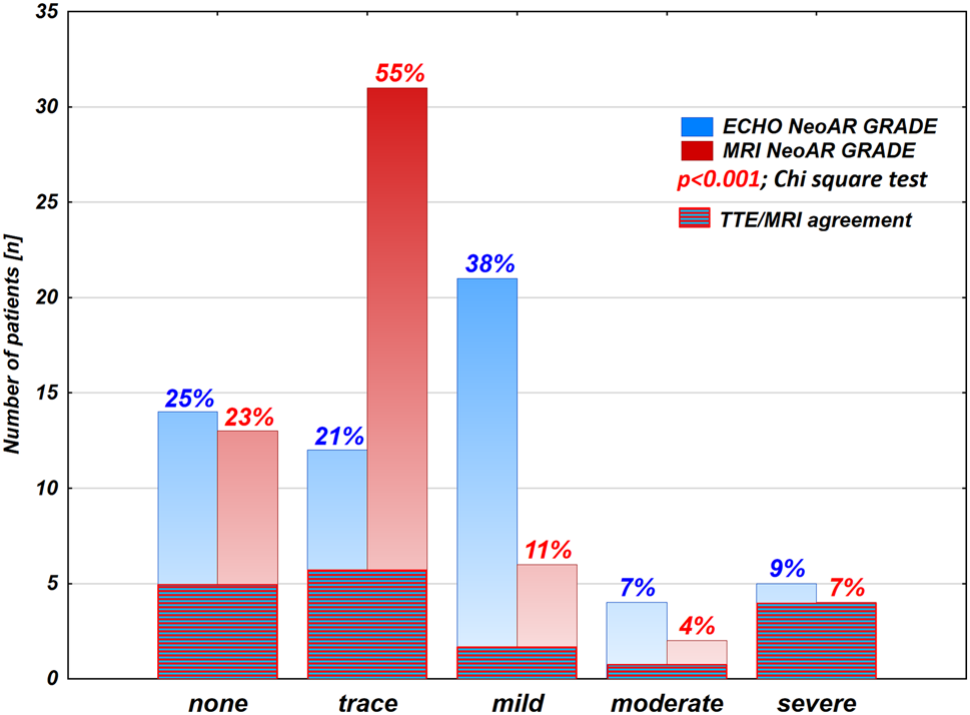
Comparison of qualitative evaluation of neoaortic insufficiency (NeoAR grade) between TTE and MRI

**Fig. 3:**
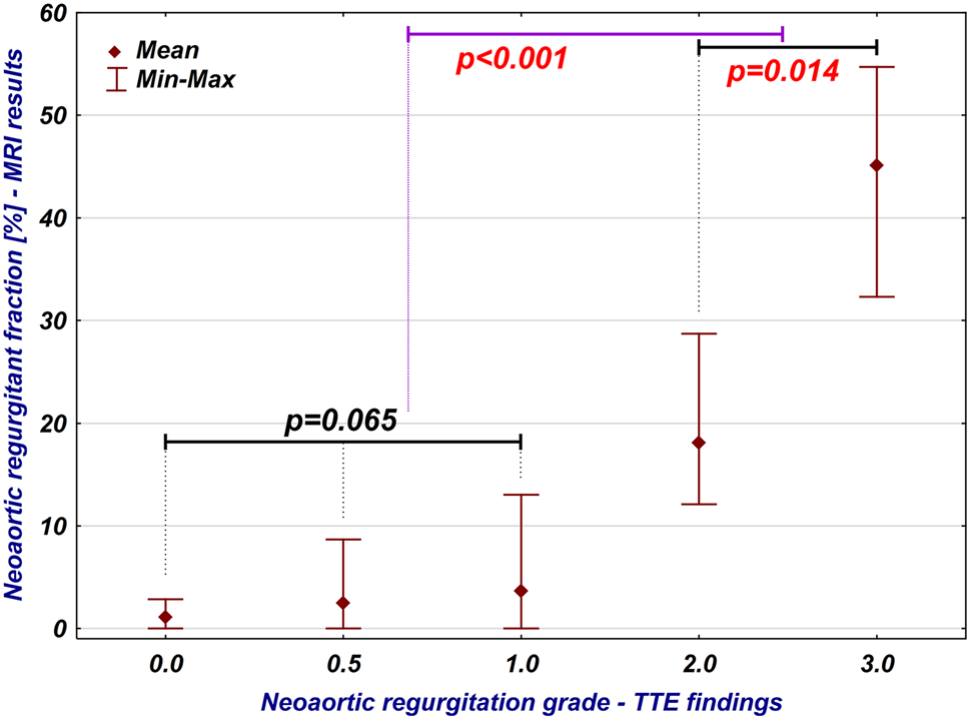
Relation between qualitative assessment of neoaortic insufficiency by TTE and quantitative data obtained via MRI (regurgitant fraction)

The median age at the time of examinations was 19.8 years (IQR 17.9–23 years). Neoaortic insufficiency was a common finding in our study group. On the basis of TTE, NeoAR was present in 75% of patients (42 out of 56). In most of the cases, it was trace or mild (12/56 patients, 21.4%, and 21/56 patients, 37.5%, respectively) and had no clinical significance. Moderate stenosis was present in 4 cases (7.1%), and 5 patients showed signs of severe stenosis (8.9%). Among this group most of the patients were examined routinely (53 patients), additionally 3 cases were qualified earlier for these studies because of severe neoaortic regurgitation. Comparing these data to the MRI results, regurgitation was found in 43 cases (76.8%) and we mostly found trace insufficiency (31/56 patients, 55.4%); mild insufficiency was found in 6 patients (10.7%), moderate was present only in 2 cases (3.6%); and 4 patients had confirmed severe regurgitation (7.1%). The differences between the qualitative assessments of neoaortic insufficiency by TTE and MRI were statistically significant (*p* < 0.001, Chi-square test, Fig. 2). The correlation between the regurgitant fraction obtained by MRI and the grade of neoaortic insufficiency on TTE is presented in Fig. 3. These two modalities correlated well in general (*R* = 0.62, *p* < 0.001, Spearman correlation test), but there were no statistically significant differences in the regurgitant fraction of the NeoAoV among patients with no, trace and mild insufficiency on echocardiography (*p* = 0.065, ANOVA, Kruskal–Wallis test). However, the difference between these patients and those with moderate and severe insufficiency was statistically significant (*p* < 0.001, Mann–Whitney *U* test).

The presence of neoaortic insufficiency was significantly correlated with bicuspid aortic valve (*p* = 0.019); in these patients, the regurgitant fraction calculated via MRI was significantly larger (median 8.7, IQR 2 to 32.3) than that in patients with tricuspid valve (median 2, IQR 0 to 4.3, *p* = 0.015).

The regurgitant fraction obtained via MRI was significantly correlated with the left ventricular end-diastolic volume (*R* = 0.29, *p* = 0.034) and the left ventricular end-diastolic volume indexed for body surface area (*R* = 0.31, *p* = 0.025).

### Neoaortic Valve

**Fig. 4:**
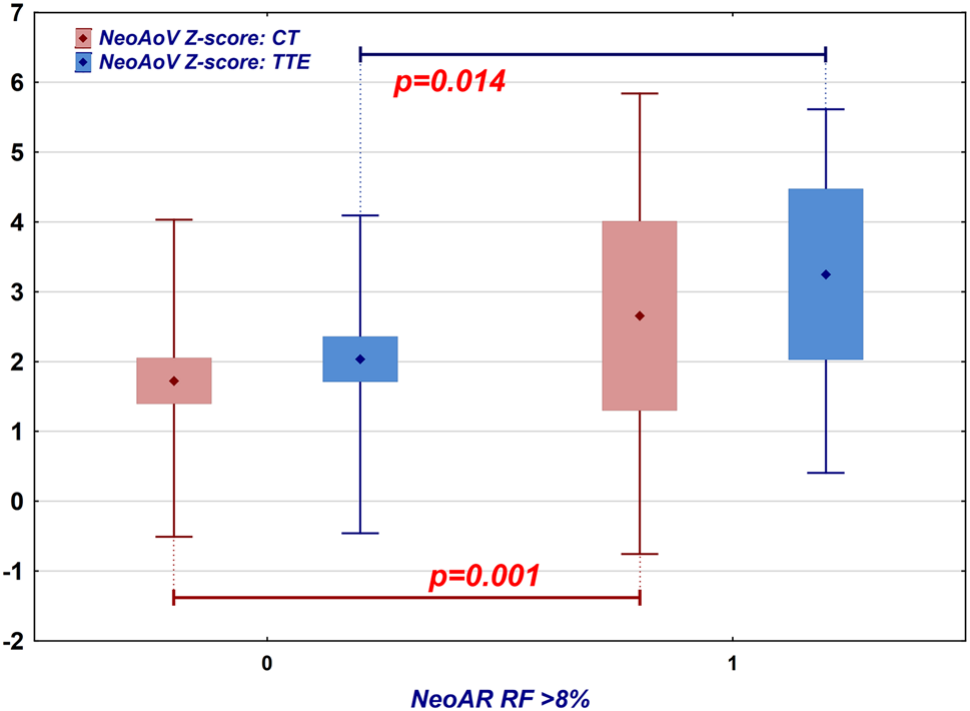
Comparison of the NeoAoV *z*-score measured by TTE (blue) and CT (red) in patients who have no or trace neoaortic insufficiency (regurgitant fraction 8% or less) and those who have at least mild insufficiency (regurgitant fraction greater than 8%). Diamond point: mean, box: 95% confidence interval, whiskers: range

In our study group, the NeoAoV diameter obtained by TTE was usually larger than the normal values; the average *z*-score was 2.27 (95% CI 1.92 to 2.64, SD 1.32). In patients who developed regurgitation qualitatively estimated as more than trace (regurgitant fraction greater than 8%, MRI), the valve diameter was significantly larger (mean *z*-score 3.25, 95% CI 2.03 to 4.47, SD 1.82) than that in patients with no or trace insufficiency (mean *z*-score 2.04, 95% CI 1.71 to 2.36, SD 1.06, *p* = 0.014, *t* test) (Fig. 4). The diameter of the valve measured via TTE was correlated significantly with the regurgitant fraction obtained via MRI (*R* = 0.32, *p* = 0.016). However, 9 out of 15 patients who had NeoAoV *z*-scores greater than 3 on TTE had no or trivial insufficiency on MRI. Among patients with regurgitant fractions, over 8% (12 patients), half had NeoAoV *Z*-scores less than 3, and 4 of them had NeoAoV *z*-scores less than 2.

The CT results were similar; the valve diameter was usually larger than the normal values (mean *z*-score: 1.92, 95% CI 1.55 to 2.3, SD 1.4). Patients with an aortic regurgitant fraction greater than 8% had a larger valve annulus (mean *z*-score: 2.66, 95% CI 1.3 to 4.01, SD 2.13) than those without insufficiency (mean *z*-score: 1.73, 95% CI 1.4 to 2.05, SD 1.1) (Fig. 4). This difference was statistically significant (*p* = 0.001, *t* test). The diameter of the NeoAoV measured on CT was significantly correlated with the MRI regurgitant fraction (*R* = 0.29, *p* = 0.032); however, 7 out of 12 patients who had a NeoAoV CT *z*-score greater than 3 had no or trivial insufficiency on MRI. Among patients with a NeoAR regurgitant fraction greater than 8% (12 patients), seven had an aortic valve CT *z*-score less than 3, and 3 had an aortic valve *z*-score less than 2.

Although the *z*-scores calculated for the echocardiographic data were higher, the diameters themselves were higher as determined by CT (CT mean 28.6, 95% CI 27.4 to 29.7, SD 4.2; TTE mean 26.2, 95% CI 25 to 27.4, SD 4.4). Both results correlated well (*r* = 0.68, *p* < 0.001, Pearson correlation test), but the observed differences were statistically significant (*p* < 0.001, paired *t* test). A significantly dilated NeoAoV (*z*-score > 3) was diagnosed in 15 cases during TTE and in 12 cases during CT. Eight patients had a coherent diagnosis of dilated aortic valve by both modalities. In most cases, the difference was related to the difference in the measurements or a significantly asymmetrical valve described by CT.

### Aortic Sinus

**Fig. 5:**
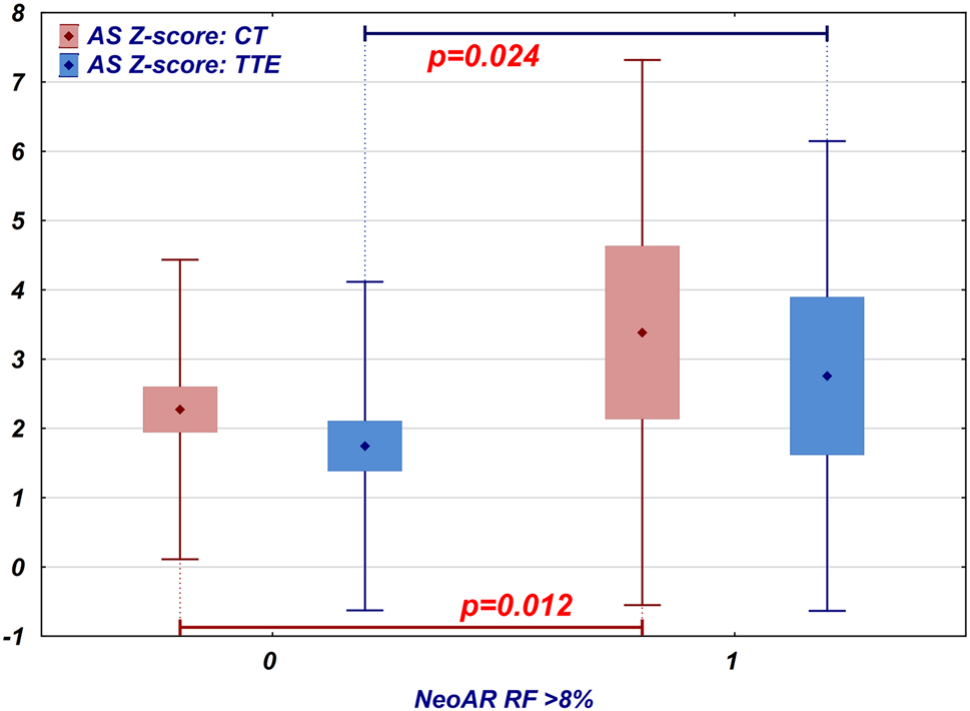
Comparison of the neoaortic sinus *z*-score measured by TTE (blue) and CT (red) in patients who have no or trace neoaortic insufficiency (regurgitant fraction 8% or less) and those who have at least mild insufficiency (regurgitant fraction greater than 8%). Diamond point: mean, box: 95% confidence interval, whiskers: range

The average diameter of the aortic sinus on echocardiography was larger than would be expected in the normal population (mean *z*-score 1.95, 95% CI 1.58 to 2.31, SD 1.35). The neoaortic sinus was significantly wider in patients with NeoAR (regurgitant fraction greater than 8%, MRI) than in those with no or trivial insufficiency (mean *z*-score 2.76, 95% CI 1.62 to 3.9, SD 1.7 vs mean *z*-score 1.74, 95% CI 1.38 to 2.1, SD 1.19, respectively, *p* = 0.024, *t* test) (Fig. 5). The aortic sinus TTE *z*-score was not significantly correlated with the regurgitant fraction calculated on MRI (*R* = 0.22, *p* = 0.108). In the group of patients with regurgitant fractions greater than 8% (12 patients), 6 had aortic sinus *z*-scores less than 3, and among them, 4 patients had *z*-scores less than 2. Eleven patients had a diagnosis of significant aortic sinus dilatation on TTE, but among them, 6 had only trace insufficiency detected via MRI.

The diameters of the aortic sinus determined by the CT examination were also larger than the reference values; the mean *z*-score was 2.51 (95% CI 2.14 to 2.88, SD 1.38). Similar to the TTE findings, a larger sinus was observed in these patients, who had insufficiency with a regurgitant fraction greater than 8% (mean *z*-score 3.38, 95% CI 2.13 to 4.63, SD 1.97 vs mean *z*-score 2.27, 95% CI 1.94 to 2.6, SD 1.08) (Fig. 5). This difference was statistically significant (*p* = 0.012, *t* test). Aortic sinus CT *Z*-scores significantly correlated with the NeoAoV regurgitant fraction obtained by MRI (*R* = 0.34, *p* = 0.011). In the group of patients with aortic sinus *z*-scores greater than 3 (16 patients), 10 patients had only trace insufficiency on MRI. In the group of patients with significant regurgitation, 6 patients had aortic sinus CT *z*-scores less than 3, and among them, 2 patients had aortic sinus *z*-scores in the normal range.

In the case of the aortic sinus, not only the calculated *z*-scores but also the diameters themselves were higher on CT than on TTE (CT mean 37.45, 95% CI 35.78 to 39.11, SD 6.22; TTE mean 35.21, 95% CI 33.66 to 36.76, SD 5.73). The results of these two modalities correlated well (*R* = 0.79, *p* < 0.001, Spearman correlation test); however, the differences were statistically significant (*p* < 0.001, Wilcoxon test). Significantly dilated aortic sinus (*z*-score > 3) was diagnosed in 11 patients by TTE and in 16 patients by CT. Nine patients had a coherent diagnosis of dilated aortic valve via both modalities.

Among anomalies associated with TGA, in our cohort, the Taussig Bing anomaly was correlated with higher aortic sinus *z*-scores for both CT (*p* = 0.031) and TTE (*p* = 0.014). Similarly, the intraoperatively confirmed significant discrepancy of the arterial valves also increased the aortic sinus *z*-scores calculated for the TTE (*p* = 0.014) and CT (*p* = 0.006) datasets.

### Sinotubular Junction

**Fig. 6:**
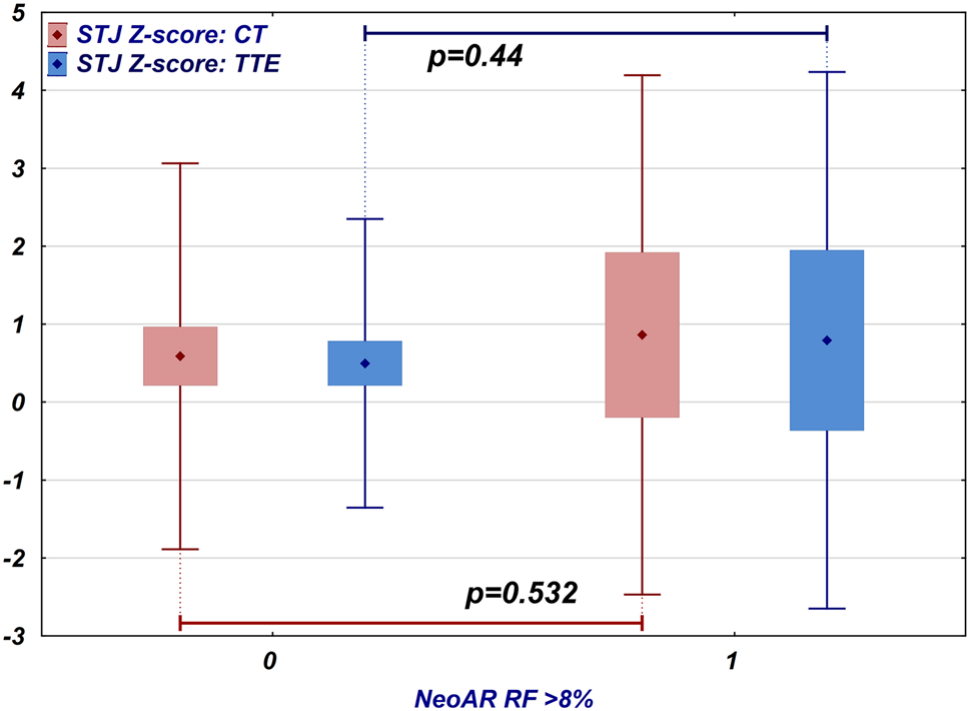
Comparison of the sinotubular junction *z*-scores measured by TTE (blue) and CT (red) in patients who have no or trace neoaortic insufficiency (regurgitant fraction 8% or less) and those who have at least mild insufficiency (regurgitant fraction greater than 8%). Diamond point: mean, box: 95% confidence interval, whiskers: range

The average diameter of the sinotubular junction obtained by TTE was close to the reference values (mean *z*-score 0.56; 95% CI 0.25 to 0.86; SD 1.12). The difference between the patients who developed NeoAR with a regurgitant fraction greater than 8% and those patients without significant insufficiency was not significantly different (mean *z*-score 0.79, 95% CI − 0.36 to 1.95, SD 1.72 vs mean *z*-score 0.5, 95% CI 0.21 to 0.77, SD 0.93, respectively, *p* = 0.44, *t* test) (Fig. 6). The results from the CT examination were consistent with the TTE findings (mean *z*-score 0.65; 95% CI 0.29 to 1; SD 1.33), and the difference between patients with and without significant regurgitation was not statistically significant (mean *z*-score 0.86, 95% CI − 0.2 to 1.92, SD 1.67 vs. mean *z*-score 0.59, 95% CI 0.21 to 0.97, SD 1.24, respectively; *p* = 0.532, *t* test) (Fig. 6). The diameter of the sinotubular was not significantly correlated with the neoaortic regurgitant fraction determined by MRI for either modality (TTE *R* = − 0.1, *p* = 0.457; CT *R* = 0.16, *p* = 0.24). The sinotubular junction diameters obtained via TTE and CT correlated well (*R* = 0.48, *p* < 0.001); however, the difference between the results was statistically significant (*p *= 0.003, Wilcoxon test); the mean difference between the CT and TTE results was 2.08 mm (95% CI 0.86 to 3.29, SD 4.44).

Among the associated defects, aortic arch anomalies were correlated significantly with the smaller sinotubular junction CT *z*-score values (*p* = 0.047).

## Discussion

Currently, NeoAoV regurgitation seems to be the most common complication after ASOs, its reason is multifactorial what implicates the different postoperative courses in different patients. The association of neoaortic insufficiency and root dilatation in this group of patients was previously described [1, 11, 12]. The surgical technique changes the root diameter at every level, thus, in most patients, the average root diameters are larger postoperatively than the normal values. Additionally, the shape and spatial position of the aortic sinus and sinotubular junction are different than those of the normal, unoperated aorta (Fig. 1a, b). The transplanted coronaries are usually placed slightly higher than their normal central position in the aortic sinuses. This is related to the fact that on average, in TGA, the anteriorly placed native aortic valve is higher than the pulmonic valve, and the coronary pattern needs to be kept as close to the original as possible. Therefore, the wider area of the aortic sinus is usually close to or above the commissural level, irrespective of the surgical technique. Transplanted coronaries may additionally widen the aortic sinus by creating the anteriorly placed convex deformations of the aortic wall. The sinotubular junction seems to have diameters closest to those in the reference range, which might be related to the slightly impaired growth potential of the sutured tissue or the pressure imparted by surrounding pulmonary arteries after the Lecompte manoeuver. The native pulmonary valve is usually larger than the aorta, so after an ASO, the average neoaortic valve diameter is larger than the normal values provided for aortic valves. In addition, the size of the valve may be impacted by the sinus diameter and histological structure of the native pulmonary valve, which has been proven to be different than that of the aortic valve [4].

The presented frequency of neoaortic valve insufficiency is quite high, but it is mostly a benign complication, as in the majority of cases, the regurgitant jet is usually trace or mild. In our dataset, TTE tended to overestimate the regurgitation grade, as shown in Fig. 2. The correlation between the MRI and TTE datasets is good, with the exclusion of trace and mild insufficiencies close to the detection limits, for which TTE was usually more sensitive and reliable. In patients with significant insufficiency, MRI quantification was definitely more reliable than TTE and is currently the gold standard for the evaluation of AR. Our cohort clearly shows that NeoAR is related to the valve and sinus diameters and occurs more frequently in patients with dilatation. Jet observation in only a few of the insufficiency cases can be explained by the root dilatation, and only a few of the significant root dilatation cases showed subsequent insufficiency of the NeoAoV. We speculate that factors other than root dilatation are sufficient to cause significant AR. From the other hand, a larger root diameter related to the initial surgical technique and disproportion between arterial valves will not necessarily result in subsequent insufficiency.

The surgical technique is frequently proposed as a potential risk factor for the development of NeoAR and aortic root dilatation [13, 14]. In our cohort, almost half of patients in the analyzed group (25 out of 56) underwent an ASO before we introduced the trap door technique. Surprisingly, there were no significant differences in the root diameters or neoaortic insufficiency between patients who underwent the punch and trap door surgeries. Additionally, the frequency of patients qualified with valve and sinus dilatation (*z*-score > 3) was similar irrespective of the surgical approach. As both techniques in our institution were used by the same surgical team and the main operator (JJM), this is evidence that both techniques influence the root size and valve function in a similar way. Of course, there is a significant difference in the reported frequency of these complications between different centers using both the same and different surgical methods of coronary transfer. Therefore, the method of coronary transplantation is important, but in our opinion, with proper experience, one can benefit from the trap door technique without increasing the risk of root dilatation or neoaortic insufficiency [14].

The root dilatation in this group of patients requires special attention not only because it may result in valve incompetence but also because of its impact on coronary arteries. Many patients with TGA have a left coronary artery placed more anteriorly in the aortic sinus, just below the pulmonary artery, with acute proximal angulation and a somewhat angulated proximal course with a tendency to reduce the cross-sectional area [5]. Looking at Fig. 1b, one can imagine that root dilatation in such a situation may lead to left coronary artery compression. Dilated roots may also have thinner walls and inappropriate structures of collagen fibers, so in some hypertensive states, such as excessive exertion or arterial hypertension, they may also result in unproportional dilatation resulting in temporal compression of such positioned coronary arteries. As these patients are relatively young, most are still asymptomatic, with normal results of routinely performed screening tests. So in our opinion, in every single patient with TGA after an ASO, we need to know the exact coronary and aortic root anatomy to make proper decisions related to the frequency of follow-up visits and recommendations related to the patient’s lifestyle and sport activities [5].

## Conclusions

Neoaortic insufficiency is quite common in patients with transposition after arterial switch procedure, but is usually a benign complication. Transthoracic echocardiography has a tendency to overestimate the severity of regurgitation compared to MRI.

Neoaortic valve and aortic sinus diameters in these group of patients are larger in relation to the normal population, and they are significantly associated with neoaortic insufficiency; however, not every case of significant regurgitation is related to root dilatation, and not every case of significant root dilatation is related to valve incompetence. The root diameters are changed similarly irrespective of the surgical technique used.
